# Adverse drug reactions in SARS-CoV-2 hospitalised patients: a case-series with a focus on drug–drug interactions

**DOI:** 10.1007/s11739-020-02586-8

**Published:** 2020-12-23

**Authors:** Giada Crescioli, Valentina Brilli, Cecilia Lanzi, Andrea Burgalassi, Alessandra Ieri, Roberto Bonaiuti, Elias Romano, Rinaldo Innocenti, Guido Mannaioni, Alfredo Vannacci, Niccolò Lombardi

**Affiliations:** 1 Department of Neurosciences, Psychology, Drug Research and Child Health, Section of Pharmacology and Toxicology, University of Florence, Florence, Italy; 2 Tuscan Regional Centre of Pharmacovigilance, Florence, Italy; 3 Toxicology Unit, Emergency Department, Careggi University Hospital, Florence, Italy; 4 Joint Laboratory of Technological Solutions for Clinical Pharmacology, Pharmacovigilance and Bioinformatics, University of Florence, Florence, Italy; 5 Internal Medicine Unit 2, Emergency Department, Careggi University Hospital, Florence, Italy; 6 Department of Experimental and Clinical Medicine, University of Florence, Florence, Italy

**Keywords:** Drug–drug interactions, COVID-19, Pharmacovigilance, Adverse drug reactions, Internal medicine

## Abstract

Due to the need of early and emergency effective treatments for COVID-19, less attention may have been paid to their safety during the global emergency. In addition, characteristics of drug–drug interaction (DDI)-related adverse drug reactions (ADRs) in COVID-19 patients have not yet been studied in depth. The aim of the present case-series study is to describe clinical and pharmacological characteristics of SARS-CoV-2 hospitalised patients, focusing on ADRs, particularly those related to DDIs. We evaluated all reports of COVID-19 medication-related ADRs collected within the COVID-19 Units of Careggi University Hospital, Florence (Italy), between January 1st and 31st May 2020. Information regarding COVID-19 medications, patients’ demographic and clinical characteristics, concomitant drugs, ADRs description and outcome, were collected. Each case was evaluated for the causality assessment and to identify the presence of DDIs. During the study period, 23 Caucasian patients (56.5% males, mean age 76.1 years) experienced one or more ADRs. The majority of them were exposed to polypharmacy and 17.4% presented comorbidities. ADRs were referred to cardiovascular, psychiatric and gastrointestinal disorders. The most frequently reported preferred term was QT prolongation (mean QT interval 496.1 ms). ADRs improved or resolved completely in 60.8% of cases. For all patients, a case-by-case evaluation revealed the presence of one or more DDIs, especially those related to pharmacokinetic interactions. Despite the small number of patients, our evidence underline the clinical burden of DDIs in SARS-CoV-2 hospitalised patients and the risk of unexpected and uncommon psychiatric ADRs.

**Supplementary Information:**

The online version contains supplementary material available at 10.1007/s11739-020-02586-8.

## Introduction

Since the outbreak of COVID-19 epidemic, clinicians started a real “gold rush” to find the best therapeutic option among those currently available for infectious and inflammatory diseases [1]. Waiting for a vaccine, the study of COVID-19 clinical characteristics proved effective in directing towards meaningful medical choices.

Three main stages represent COVID-19 infection clinical course. In the first phase, the virus replicates within the host cells and patients may experience symptoms as dry cough, fever, and general weakness and malaise [2]. In the second phase, the progression of the disease is characterised by the development of a bilateral interstitial pneumonia and by morphological changes in host’s lungs [3]. Respiratory symptoms, which could be stable in the first phase of pneumonia, could worsen, due to both direct effects of the virus and host’s immune response, leading to clinical instability and severe hypoxemia [4]. Only in a limited number of cases (third phase), patients experience a “cytokine storm” and following hyper-inflammatory state, with local and systemic consequences [5]. Among them, at the lung level, are arterial and venous vasculopathy, with thrombosis of the small vessels and evolution towards serious and sometimes permanent lung lesions. A progressive alteration of inflammatory and coagulation parameters, such as C-reactive Protein (PCR), ferritin, pro-inflammatory cytokines, consumption of clotting factors, and increased levels of the fragments of fibrin degradation (D-dimer), have been observed [6, 7].

In this complex scenario, therapeutic strategies have focused on viral growth containment in the first and second phases of the disease, and on inflammation and coagulation control, in the second and third phases (Table 1) [8]. When COVID-19 pandemic spread to Italy, the Italian Medicines Agency (AIFA) approved the off-label use of the antiviral combinations lopinavir/ritonavir and darunavir/cobicistat, the use of antimalarials chloroquine and hydroxychloroquine (HCQ), the antibiotic azithromycin, and the anticoagulant enoxaparin [9]. Clinical experience also suggested the use of tocilizumab, a humanised monoclonal antibody against interleukin (IL)-6 receptor [10].

**Table 1:** Indication of use and mechanism of action of principal SARS-CoV-2 medications in Italy

SARS-CoV-2 infection phase	Medication	Indication	Mechanism of action	AIFA authorization
Phase I and II Viral growth containment	Darunavir/cobicistat Lopinavir/ritonavir	HIV treatment	Inhibition of viral replication by the binding and inactivation of the 3CLpro and PL2pro proteases	Off-label use restricted to RCTs (last update July 17th, 2020)
	Hydroxychloroquine Chloroquine	Antimalarials, antirheumatics	Increasing of endosomal pH crucial for virus-cell fusion	Off-label use restricted to RCTs (last update July 22^nd^, 2020)
	Remdesivir*	Ebola virus	In vitro and in vivo activity against SARS-CoV-2, MERS-CoV and SARS-CoV	Compassionate use
	Ribavirin*	Chronic HCV and RSV infections	Guanosine analogue that interferes with the replication of RNA and DNA viruses	Compassionate use
Phase III Inflammation and coagulation control	Azithromycin	Antibacterial for systemic use	Downregulation of adhesion molecules of cell surface, reduction of pro-inflammatory cytokines production	Authorised out of RCTs only in SARS-CoV-2 positive adult patients with bacterial infections (last update May 5th, 2020)
	Canakinumab*	Arthritis, autoinflammatory fever, Still’s disease (IL-1 β antibody)	Reduction of SARS-CoV-2-induced pneumonia and inflammation	Compassionate use
	Enoxaparin	Prophylaxis of venous thromboembolism	Containment of thrombotic phenomena from the pulmonary circulation	Off-label use
	Tocilizumab	RA (IL-6 receptor antibody)	Reduction of SARS-CoV-2-induced pneumonia and inflammation	Off-label use
	Ruxolitinib*	Myelofibrosis (inhibitor of JAK1 and JAK2 kinases)	Reduction of SARS-CoV-2-induced pneumonia and inflammation	Compassionate use
	Solnatide*	Pseudo-hypoaldosteronism 1B	In study to treat pulmonary permeability oedema in Austria and Germany	Compassionate use

*AIFA* Italian Medicines Agency, *CoV* coronavirus, *HCV* hepatitis virus C, *HIV* Human Immunodeficiency Viruses, *MERS* Middle East Respiratory Syndrome, *RA* rheumatoid arthritis, *RCT* randomised clinical trial, *RSV* respiratory syncytial virus, *SARS* severe acute respiratory syndrome

*Compassionate use

However, due to the need of early and emergency effective treatments for COVID-19, less attention may have been paid to their safety during the global emergency. From the beginning of COVID-19 pandemic, concerns about HCQ efficacy and safety raised [11] and subsequently AIFA suspended its use in SARS-CoV-2 patients out of clinical trials on May 29^th^, 2020 [12]. Moreover, based on available evidence concerning the efficacy and safety of fixed associations darunavir/cobicistat and lopinavir/ritonavir, AIFA also suspended their use out of clinical trials on July 17th 2020 [13, 14].

Prevalence of adverse drug reactions (ADRs) in COVID-19 patients has not yet been deeply evaluated, but results from observational studies suggest that its frequency could be high in this population [15]. The majority of ADRs includes gastrointestinal and liver system disorders. Nevertheless, potential harmful ADRs should be closely monitored, and pharmacovigilance monitors’, toxicologists’ and clinical pharmacologists’ support in COVID-19 Units should be carefully considered to better manage COVID-19 therapies [16]. In particular, good quality information regarding drug–drug interaction (DDI)-related ADRs are still lacking.

In this context, the aim of the present case-series study is to describe clinical and pharmacological characteristics of SARS-CoV-2 hospitalised patients, focusing on ADRs, particularly those related to DDIs.

## Methods

In the present observational study, we considered and evaluated all reports of suspected ADRs collected in the COVID-19 Units of Careggi University Hospital, Florence (Italy), between January 1st and 31st May, 2020. All suspected ADRs were collected from the clinical charts after a consultation performed by the Toxicology Unit on request of clinicians working in COVID-19 Units.

Following the Italian pharmacovigilance legislation [17], a multidisciplinary team composed by experts in pharmacovigilance (GC, AV, and NL) and clinical toxicology (VB, CL, AB, AI, and GM) provided their consultation and filled out the specific report form [18, 19], collecting information on: (1) patients’ demographic characteristics (age, gender, ethnic group); (2) patients’ clinical status; (3) suspected drugs and concomitant medications (for each one, administration route, therapy duration, dosages, and therapeutic indication were recorded); (4) ADR description; and (5) ADR outcome (improvement, complete resolution, unchanged or worsened event, resolution with sequelae, and death). A “suspected drug” is defined as a drug which is potentially associated with the observed ADR, while a “concomitant medication” is a drug the patient is exposed to at the time of ADR occurrence. A concomitant medication may not necessarily be associated to the ADR.

For each case included in the analysis, the experts performed a medical evaluation to assess the causality relationship between the suspected drugs and their related ADRs according to the Naranjo’s scale [20]. Moreover, each case was evaluated with the aim of identifying the presence of DDIs, which may have contributed to ADRs. DDIs were identified using two different validated tools: (1) the open access Drug Interaction Checker [21], and (2) the drug interaction software IBM Micromedex^®^ (Thomson Reuters Healthcare Inc., Greenwood Village, Colorado, United States), available online with restricted access. As reported in the IBM Micromedex^®^ [22] and Drug Interaction Checker [21] tools, DDIs were classified as mild, moderate, or major, depending on their clinical impact on patient.

Suspected drugs and concomitant medications were classified according to the Anatomical Therapeutic Chemical (ATC) classification system. ADR description according to diagnosis and symptoms was coded using the Medical Dictionary for Regulatory Activities (MedDRA) and organised by Preferred Term (PT) [23, 24].

Data are presented as number and percentages or, for continuous variables, as mean and standard deviation (SD).

## Results

Between January 1st and May 31st, 2020, among patients hospitalised in the COVID-19 Units of Careggi University Hospital, Florence (Italy), clinicians requested a consultation for a total of 23 patients who experienced 1 or more ADRs.

All patients were Caucasian with a mean age of 76.1 (SD ± 14.4) years. 56.5% of them were males, and the majority (82.6%) were elderly (age ≥ 65 years). All patients (*n* = 23) were exposed to more than one suspected drug. In 56.5% (*n* = 13) of cases, more than three suspected medications were reported. Overall, 69% (*n* = 16) of patients were treated with more than six concomitant medications, and 17.4% (*n* = 4) presented more than six comorbidities. ADRs improved or resolved completely in 60.8% of cases, and were probably or possibly related to suspected drugs for all patients (Table 2).

**Table 2:** Patients’ characteristics

Cases characteristics	No. of cases *N* = 23 (%)
Patient age, years	
19–64	4 (17.4)
65–79	5 (21.7)
≥ 80	14 (60.9)
Mean ± standard deviation	76.1 ± 14.40
Gender	
Male	13 (56.5)
Female	10 (43.5)
No. of suspected drugs	
2	10 (43.5)
3	8 (34.8)
≥ 4	5 (21.7)
No. of concomitant medications	
None	3 (13.2)
1–5	4 (17.4)
6–10	11 (47.7)
≥ 11	5 (21.7)
No. of comorbidities	
None	5 (21.7)
1–5	14 (60.9)
≥ 6	4 (17.4)
Outcome	
Improvement	14 (60.8)
Death	5 (21.7)
Unchanged or worsened event	3 (13.2)
Complete resolution	1 (4.3)
Causality assessment	
Certain	–
Probable	17 (73.9)
Possible	6 (26.1)
Not classifiable	–
QT prolongation (19 patients), ms	
Mean ± standard deviation	496.1 ± 36.64

According to Careggi University Hospital’s standard procedures in use at the time of the survey, all SARS-CoV-2 hospitalised patients were administered with HCQ 800 mg for the first day, followed by 400 mg/daily for 5–7 days, alone or in association to lopinavir/ritonavir 400/100 mg twice/daily for 7–14 days. Clinicians could add azithromycin (500 mg for the first day, followed by 250 mg/daily for 3–4 days) or darunavir/cobicistat (800/150 mg/daily) to HCQ, according to patient’s needs. Patients received enoxaparin at the dosage of 4000 or 8000 International Unit (IU)/daily based on their body weight and the presence of thromboembolic risk factors, such as Sepsis-Induced Coagulopathy (SIC) score ≥ 4, neoplasia, central venous catheter, d-dimer levels > 1000 ng/mL, Continuous Positive Airway Pressure (CPAP) or history of venous thromboembolism.

Table 3 shows a case-by-case description of evaluated ADR reports. One patient presented an elevation of transaminases, 3 patients experienced gastrointestinal (GI) ADRs (nausea, diarrhoea, vomiting and GI pain), 18 patients showed cardiovascular (CV) ADRs (ECG QT prolongation), 1 patient presented a prolongation of the QT interval along with symptoms of major depression, and 1 patient reported psychotic symptoms. The 19 patients who experienced an ECG QT prolongation showed a mean prolongation of QT interval of 496.1 (SD ± 14.4) ms (Table 2).

**Table 3:** Case-by-case clinical description of all evaluated reports

Case	Age (years)	M/F	Adverse drug reactions (PT)	Outcome	Suspected drugs	Concomitant medications	Comorbidities
1	36	F	Nausea, vomiting, abdominal pain, drug level modification	Improvement	Tacrolimus Darunavir/cobicistat	None	Renal transplant
2	80	M	ECG QT prolonged, atrial flutter, hemiplegia, hypokalaemia, major depression	Improvement	HCQ Risperidone	Sertraline, olanzapine, lorazepam, ceftriaxone, enoxaparin, lopinavir/ritonavir*, and darunavir/cobicistat*	None
3	61	M	Hypertransaminasemia	Improvement	Lopinavir/ritonavir Darunavir/cobicistat Tocilizumab HCQ	Azithromycin, bisoprolol, pantoprazole, enoxaparin, lorazepam, and atorvastatin	Atrial fibrillation
4	52	F	Diarrhoea, vomiting	Complete resolution	Darunavir/cobicistat HCQ Azithromycin	None	None
5	78	F	ECG QT prolonged, hypokalaemia	Improvement	HCQ Lopinavir/ritonavir	Darunavir/cobicistat*	Cardiomyopathy and pacemaker
6	84	M	ECG QT prolonged, vomiting	Improvement	HCQ Lopinavir/ritonavir Azithromycin	None	Pancreatic lesions
7	56	M	ECG QT prolonged	Improvement	HCQ Lopinavir/ritonavir Azithromycin	Darunavir/cobicistat*, enoxaparin, acetylsalicylic acid, candesartan, losartan, betamethasone, and amoxicillin	Hypertension
8	93	F	ECG QT prolonged	Improvement	HCQ Citalopram	Simvastatin, levothyroxine, bromazepam, trazodone, enoxaparin, azithromycin, ceftriaxone	None
9	82	F	ECG QT prolonged	Improvement	HCQ Lopinavir/ritonavir Azithromycin	Sevelamer, pantoprazole, bisoprolol, furosemide, methylprednisolone, piperacillin/tazobactam, linezolid, and epoetin alpha	Renal failure, anaemia, hypertension
10	80	M	ECG QT prolonged	Unchanged event	HCQ Sertraline Azithromycin	Valsartan, esomeprazole, pantoprazole, lorazepam, amlodipine, mesalamine, tamsulosin, loperamide, piperacillin/tazobactam, acetylsalicylic acid, enoxaparin, and furosemide	None
11	67	F	ECG QT prolonged	Unchanged event	HCQ Lopinavir/ritonavir Azithromycin Haloperidol Levomepromazine Zuclopenthixol	Valproic acid, atorvastatin, lorazepam, and fondaparinux	Paranoid schizophrenia, hypercholesterolemia
12	85	M	ECG QT prolonged	Unchanged event	Lopinavir/ritonavir HCQ	Piperacillin/tazobactam, magnesium sulphate, linezolid, fluticasone propionate/vilanterol, bisoprolol, pantoprazole, thiamazole, allopurinol, acetylsalicylic acid, ursodeoxycholic acid, calcium carbonate, and calcitriol	Renal failure, hyperthyroidism, BPH, heart failure
13	70	F	ECG QT prolonged	Death	HCQ Darunavir/cobicistat Azithromycin Amiodarone	Warfarin, furosemide, potassium chloride, atorvastatin, amoxicillin/clavulanate, acetaminophen, enoxaparin, ruxolitinib, and aclidinium bromide	COPD, hypothyroidism, atrial fibrillation, renal failure
14	86	M	ECG QT prolonged	Improvement	HCQ Lopinavir/ritonavir	Furosemide, apixaban, atorvastatin, nitroglycerine, clonidine, potassium chloride, darunavir/cobicistat*, and enoxaparin	None
15	93	M	ECG QT prolonged	Death	Lopinavir/ritonavir HCQ Azithromycin	Ceftriaxone, furosemide, bisoprolol, tamsulosin, dutasteride, ramipril, enoxaparin, insulin, gliclazide, vildagliptin, metformin, perindopril, barnidipine, and simvastatin	Diabetes, hypertension, BPH, vascular encephalopathy, lower limbs obliterating arteriopathy
16	80	M	ECG QT prolonged	Death	Lopinavir/ritonavir HCQ Azithromycin Haloperidol	Verapamil, lansoprazole, acetylsalicylic acid, macrogol, and enoxaparin	Alzheimer and Parkinson’s diseases, hypertension, right carotid stenosis, renal failure, hepatic steatosis
17	85	M	ECG QT prolonged, hypokalaemia	Improvement	HCQ Magnesium sulphate	Tamsulosin, bisoprolol, warfarin, atorvastatin, bicalutamide, rabeprazole, meropenem, vancomycin, and potassium chloride	Hypertension, hypercholesterolemia, neuropathic pain, prostatic cancer, mitral valve intervention, hypokalaemia and hypomagnesemia
18	71	M	ECG QT prolonged	Improvement	HCQ Darunavir/cobicistat	Simvastatin, allopurinol, olmesartan, nebivolol, rivaroxaban, and potassium chloride	Hypertension, dyslipidaemia, atrial fibrillation, pulmonary emphysema, obesity
19	89	M	ECG QT prolonged	Initial improvement of QT prolongation and death	Lopinavir/ritonavir HCQ Azithromycin Citalopram	Pantoprazole, bisoprolol, clopidogrel, atorvastatin, quetiapine, promazine, piperacilline/tazobactam, enoxaparin, and olanzapine	Mild mental retardation, Parkinson's disease, past severe head injury with subdural hematoma, chronic dysphagia, thrombocytopenia, anaemia, hypertension, dyslipidaemia, chronic renal failure
20	65	M	Psychosis, agitation, delirium, aggressiveness	Improvement	HCQ Darunavir/cobicistat Tocilizumab	Dutasteride, allopurinol, propofol, risperidone, valproic acid, clonazepam, ramipril, amlodipine, midazolam, spironolactone, dexmedetomidine, and olanzapine	Overweight, hyperuricemia, prostatic hypertrophy
21	81	F	ECG QT prolonged	Improvement	HCQ Darunavir/cobicistat	Thiamazole, pantoprazole, folic acid, potassium chloride, bisoprolol, furosemide, warfarin, potassium canrenoate, alprazolam, enoxaparin and iron, vitamin C and vitamin B_12_ supplementation	Atrial fibrillation, hypertension, hyperthyroidism, osteoporosis, mitral valve disease
22	89	F	ECG QT prolonged	Death	HCQ Trazodone	Ceftriaxone, methylprednisolone, acetylsalicylic acid, acetaminophen/codeine, alprazolam, omeprazole, valproic acid, morphine, enoxaparin, calcium and vitamin D_3_ supplementation	Cognitive impairment, chronic subcortical vascular encephalopathy, kidney heteroplasia, hypertension, osteoporosis, osteoarthritis, anxiety-depressive syndrome
23	88	F	ECG QT prolonged	Improvement	HCQ Darunavir/cobicistat Haloperidol	Promazine, enoxaparin, and potassium chloride	Alzheimer’s disease

*BPH* benign prostatic hyperplasia, *COPD* chronic obstructive pulmonary disease, *ECG* electrocardiogram, *F* female, *HCQ* hydroxychloroquine, *M* male

*This medication was not administered simultaneously with the suspected drugs. In particular, the patient was not exposed to this medication at the time of ADR occurrence

Table 4 shows all moderate and major DDIs observed in our sample between COVID-19 treatments and patients’ concomitant medications. The application of tools for interactions revealed that all patients presented at least one DDI. Among 82 different DDIs, 53 (64.4%) were moderate, and 32 (39%) increased the risk of QT prolongation. Many others DDIs (*n* = 112) were identified between medications other than COVID-19 treatments (Supplementary Table 1).

**Table 4:** Major and moderate drug–drug interactions between COVID-19 treatments and patients’ concomitant medications

Interacting drugs	Interacting active principles	Interaction severity	Interaction effects
COVID-19 medication with other COVID-19 therapies	HCQ–azithromycin	Moderate	Increased risk of QT ECG prolongation
	HCQ–lopinavir/ritonavir	Major	Increased risk of QT ECG prolongation
	HCQ–tocilizumab	Moderate	Increased risk of peripheral neuropathy
	Lopinavir/ritonavir–azithromycin	Moderate	Increased risk of QT ECG prolongation
	Lopinavir/ritonavir–darunavir/cobicistat	Moderate	Altered blood levels and effects of both medications due to darunavir/cobicistat inhibition and lopinavir inhibition or induction of CYP450 3A4
COVID-19 medication with CNS medications	Azithromycin–citalopram	Major	Increased risk of QT ECG prolongation
	Azithromycin–haloperidol	Major	Increased risk of QT ECG prolongation
	Azithromycin–olanzapine	Moderate	Increased risk of QT ECG prolongation
	Azithromycin–promazine	Moderate	Increased risk of QT ECG prolongation
	Azithromycin–quetiapine	Moderate	Increased risk of QT ECG prolongation
	Azithromycin–trazodone	Moderate	Increased risk of QT ECG prolongation
	Darunavir/cobicistat–alprazolam	Moderate	Increased blood levels of alprazolam due to darunavir/cobicistat inhibition of CYP450 3A4
	Darunavir/cobicistat–clonazepam	Moderate	Increased blood levels of clonazepam due to darunavir/cobicistat inhibition of CYP450 3A4
	Darunavir/cobicistat–midazolam	Major	Increased blood levels of midazolam due to darunavir/cobicistat inhibition of CYP450 3A4
	Darunavir/cobicistat–risperidone	Moderate	Increased blood levels of risperidone to darunavir/cobicistat inhibition of CYP450 3A4
	Darunavir/cobicistat–sertraline	Moderate	Decreased blood levels and effects of sertraline
	Enoxaparin–citalopram	Moderate	Increased risk of bleeding
	Enoxaparin–sertraline	Moderate	Increased risk of bleeding
	HCQ–citalopram	Major	Increased risk of QT ECG prolongation
	HCQ–codeine	Moderate	Increased risk of codeine reduced efficacy
	HCQ–haloperidol	Major	Increased risk of QT ECG prolongation
	HCQ–olanzapine	Moderate	Increased risk of QT ECG prolongation
	HCQ–promazine	Major	Increased risk of QT ECG prolongation
	HCQ–quetiapine	Major	Increased risk of QT ECG prolongation
	HCQ–risperidone	Moderate	Increased risk of QT ECG prolongation
	HCQ–sertraline	Moderate	Increased risk of QT ECG prolongation
	HCQ–trazodone	Major	Increased risk of QT ECG prolongation
	Lopinavir/ritonavir–citalopram	Major	Increased risk of QT ECG prolongation
	Lopinavir/ritonavir–haloperidol	Major	Increased risk of QT ECG prolongation and increased blood levels of haloperidol due to lopinavir/ritonavir inhibition of CYP450 2D6
	Lopinavir/ritonavir–olanzapine	Moderate	Increased risk of QT ECG prolongation
	Lopinavir/ritonavir–promazine	Moderate	Increased risk of QT ECG prolongation
	Lopinavir/ritonavir–quetiapine	Major	Increased blood levels of quetiapine due to lopinavir/ritonavir inhibition of CYP450 3A4 and increased risk of QT ECG prolongation
	Lopinavir/ritonavir–risperidone	Moderate	Increased risk of QT ECG prolongation
	Lopinavir/ritonavir–sertraline	Moderate	Increased risk of QT ECG prolongation
	Lopinavir/ritonavir–valproic acid	Moderate	Reduced blood levels of valproate due to lopinavir/ritonavir induction of glucuronosyltransferases
	Tocilizumab–amlodipine	Moderate	Decreased blood levels of amlodipine
	Tocilizumab–clonazepam	Moderate	Decreased blood levels of clonazepam
	Tocilizumab–midazolam	Moderate	Decreased blood levels of midazolam
COVID-19 medication with CV medications	Azithromycin–atorvastatin or simvastatin	Moderate	Increased blood levels of atorvastatin and increased risk of liver damage and myopathy due to azithromycin inhibition of CYP450 3A4
	Azithromycin–sertraline	Moderate	Increased risk of QT ECG prolongation
	Azithromycin–warfarin	Moderate	Increased risk of bleeding
	Darunavir/cobicistat–amiodarone	Major	Increased risk of QT ECG prolongation due to darunavir/cobicistat inhibition of CYP450 3A4
	Darunavir/cobicistat–amlodipine	Moderate	Increased blood levels of amlodipine due to darunavir/cobicistat inhibition of CYP450 3A4
	Darunavir/cobicistat–apixaban	Major	Increased blood levels of apixaban and increased risk of bleeding due to darunavir/cobicistat inhibition of CYP450 3A4 and P-glycoprotein
	Darunavir/cobicistat–atorvastatin or simvastatin	Major	Increased blood levels of atorvastatin and increased risk of liver damage and myopathy due to darunavir/cobicistat inhibition of CYP450 3A4
	Darunavir/cobicistat–nebivolol	Moderate	Increased blood levels of bisoprolol due to darunavir/cobicistat inhibition of CYP450 3A4
	Darunavir/cobicistat–rivaroxaban	Major	Increased blood levels of rivaroxaban and increased risk of bleeding due to darunavir/cobicistat inhibition of CYP450 3A4
	Darunavir/cobicistat–warfarin	Moderate	Altered blood levels of warfarin
	Enoxaparin–amiodarone	Major	Increased risk of bleeding
	Enoxaparin–apixaban	Major	Increased risk of bleeding
	Enoxaparin–clopidogrel	Major	Increased risk of bleeding
	Enoxaparin–losartan or valsartan	Moderate	Increased risk of hyperkalaemia
	Enoxaparin–perindopril	Moderate	Increased risk of hyperkalaemia
	Enoxaparin–warfarin	Major	Increased risk of bleeding
	HCQ–amiodarone	Major	Increased risk of QT ECG prolongation
	HCQ–atorvastatin or simvastatin	Moderate	Increased risk of peripheral neuropathy
	Lopinavir/ritonavir–apixaban	Major	Increased blood levels of apixaban and increased risk of bleeding due to ritonavir lopinavir/inhibition of CYP450 3A4 and P-glycoprotein
	Lopinavir/ritonavir–atorvastatin or simvastatin	Major	Increased blood levels of atorvastatin and increased risk of liver damage and myopathy due to lopinavir/ritonavir inhibition of CYP450 3A4
	Lopinavir/ritonavir–bisoprolol	Moderate	Increased risk of PR ECG prolongation
	Lopinavir/ritonavir–verapamil	Moderate	Increased risk of hypotension and of PR ECG prolongation
	Tocilizumab–atorvastatin	Moderate	Decreased levels of atorvastatin
COVID-19 medication with other medications	Azithromycin–loperamide	Moderate	Increased risk of QT ECG prolongation
	Darunavir/cobicistat–betamethasone	Moderate	Increased blood levels of betamethasone due to darunavir/cobicistat inhibition of CYP450 3A4
	Darunavir/cobicistat–clopidogrel	Major	Decreased efficacy of clopidogrel due to darunavir/cobicistat inhibition of CYP450 3A4/5, 1A2, 2B6
	Darunavir/cobicistat–dutasteride	Moderate	Increased blood levels of dutasteride
	Darunavir/cobicistat–ruxolitinib	Major	Increased blood levels of ruxolitinib due to darunavir/cobicistat inhibition of CYP450 3A4
	HCQ–bicalutamide	Moderate	Increased risk of QT ECG prolongation
	HCQ–fluticasone propionate/vilanterol	Moderate	Increased risk of QT ECG prolongation
	HCQ–gliclazide	Moderate	Increased risk of hypoglycaemia
	HCQ–linezolid	Moderate	Increased risk of neuropathy
	HCQ–loperamide	Moderate	Increased risk of QT ECG prolongation
	Lopinavir/ritonavir–betamethasone	Moderate	Increased blood levels of betamethasone due to lopinavir/ritonavir inhibition of CYP450 3A4
	Lopinavir/ritonavir–clopidogrel	Major	Decreased efficacy of clopidogrel due to lopinavir/ritonavir inhibition of CYP450 3A4/5, 1A2, 2B6
	Lopinavir/ritonavir–dutasteride	Moderate	Increased blood levels of dutasteride due to lopinavir/ritonavir inhibition of CYP450 3A4
	Lopinavir/ritonavir–fluticasone propionate/vilanterol	Major	Increased exposure to fluticasone propionate and vilanterol due to lopinavir/ritonavir inhibition of CYP450 3A4 and increased risk of QT ECG prolongation
	Lopinavir/ritonavir–gliclazide	Moderate	Increased risk of hyperglycaemia
	Lopinavir/ritonavir–insulin	Moderate	Increased risk of insulin inefficacy
	Lopinavir/ritonavir–levothyroxine	Moderate	Reduced blood levels of thyroxine due to lopinavir/ritonavir induction of glucuronosyltransferase
	Lopinavir/ritonavir–metformin	Moderate	Increased risk of hyperglycaemia
	Lopinavir/ritonavir–methylprednisolone	Major	Increased blood levels of methylprednisolone due to lopinavir/ritonavir inhibition of CYP450 3A4
	Lopinavir/ritonavir–tamsulosin	Major	Increased blood levels of tamsulosin due to lopinavir/ritonavir inhibition of CYP450 3A4 and 2D6; increased risk of hypotension and priapism
	Lopinavir/ritonavir–vildagliptin	Moderate	Increased risk of hyperglycaemia

*CNS* central nervous system, *CV* cardiovascular, *ECG* electrocardiogram, *HCQ* hydroxychloroquine

## Discussion

This study aimed to describe the clinical and pharmacological characteristics of SARS-CoV-2 hospitalised patients who experienced one or more ADRs, with a focus on DDIs. Based on the evidence herein reported, the majority of ADRs occurred in elderly patients exposed to polypharmacy.

Off-label drug utilisation and DDIs are well known causes of ADRs in the general population [25], and the risk of DDI-related ADRs typically increases in patients exposed to c drugs, such as those aged ≥ 65 years [24]. In the case of COVID-19 pandemic, lacking of specific pharmacological treatments forced clinicians and regulatory agencies to resort to currently available drugs. Thus, off-label drug utilisation could not be avoided. On the contrary, a patient’s complete anamnesis, in particular regarding comorbidities and concomitant medications, may help in avoiding the occurrence of ADRs, often due to DDIs.

In our sample, according to the case-by-case clinical evaluation, most frequently reported ADRs were cardiac, psychiatric and nervous system, and gastrointestinal and hepatic disorders.

### Cardiac disorders

We observed 19 cases of CV ADRs, in particular “QT prolongation”. In these cases, the most frequently reported suspected COVID-19 medications were HCQ, azithromycin, lopinavir/ritonavir and darunavir/cobicistat, which are, based on their pharmacokinetic and pharmacodynamic properties, commonly associated to CV events.

HCQ acute toxicity occurs most frequently when therapeutic or high doses are administered rapidly through parenteral routes. HCQ doses of > 5 g given parenterally usually are fatal. Toxic manifestations relate primarily to the CV system, including hypotension, suppressed myocardial function, arrhythmias, and eventual cardiac arrest. Due to its long elimination half-life (> 40 days) [26], prolonged treatment with high doses may also cause ADRs such as widening of the QRS interval, and T-wave abnormalities. In fact, it is well known that HCQ inhibits human ether-a-go-go-related gene (hERG) potassium channels. Inhibition of hERG can block the outward flow of potassium, which leads to intracellular accumulation of potassium and ventricular repolarization and results in QT prolongation and torsade de pointes (TdP) [27]. These complications usually disappear shortly after the drug withdrawal. HCQ may also inhibit CYP2D6, interacting with a variety of different COVID-19 and non-COVID-19 medications.

The macrolide azithromycin is a weak inhibitor of CYP3A4. In this case, in connection with its effect on QT prolongation, the potential for DDIs is associated to azithromycin pharmacodynamic characteristics. As such, caution should always be observed when combining azithromycin with other molecules that increase the QT interval, such as HCQ. In particular, QT prolongation seems to be significantly higher in patients who received the two medications concomitantly [28]. The exact mechanism by which azithromycin and other macrolides prolong the QT interval is through a blockade of the rapid component, IKr, of the delayed rectifier potassium current IK, which is encoded by the hERG [29], similarly to HCQ.

Special caution must be used when administering protease inhibitors, such as lopinavir/ritonavir, due to their potential of inducing QT interval prolongation, particularly when used in combination with other pro-arrhythmic medications, such as HCQ and azithromycin. In fact, lopinavir/ritonavir may increase concentrations of the co-administered medicinal products and this may result in an increase of their associated cardiac ADRs. This can be explained by the ability of lopinavir/ritonavir to modulate enzymes, in particular CYP3A4 and P-glycoprotein (P-gp). Lopinavir/ritonavir may also inhibit BCRP and OATP1B1 transporters [30]. These pharmacokinetic characteristics were also observed for darunavir/cobicistat [31], leading to a comparable profile in terms of DDIs and potentially related CV ADRs.

In general, attention should be exercised when COVID-19 treatments are combined with drugs known to increase the PR or QT intervals, as they also cause conduction and repolarization disorders by themselves. Considering that QT prolongation could be an asymptomatic and potentially fatal event, it should be always strictly monitored. The risk factors for QT prolongation and TdS are female sex, older age, heart disease, exposure to QT interval prolonging drugs or metabolic inhibitors, bradycardia, and electrolyte disturbance [28]. The cornerstone of the management of acquired QT prolongation includes the identification and discontinuation of any suspected drug and the prompt correction of any metabolic abnormalities [32]. Short-term treatment includes the administration of intravenous magnesium sulphate and potassium chloride to manage a possible hypomagnesemia or hypokalemia. Thus, ECG and serum potassium levels should be frequently checked.

### Psychiatric and nervous system disorders

We observed two cases of psychiatric disorders, in particular “major depression syndrome” and “psychotic crisis”, accompanied by agitation, delirium, and aggressiveness. In these cases, the suspected COVID-19 medications were HCQ, darunavir/cobicistat, and, for one patient, tocilizumab. Considering the presence of co-administered antidepressants, antipsychotics, and hypnotic and sedative agents, for these patients a COVID-19 drug-related reduction of the activity of central nervous system medications could not be excluded. At the same time, psychological and social distress linked to COVID-19 infection should be taken into consideration [33, 34]. In fact, a recent systematic review and meta-analysis confirmed that SARS-CoV-2 might cause depression, anxiety, neuropsychiatric syndromes, and delirium in a significant proportion of patients in the acute stage [35]. Of notice, psychiatric ADRs are not commonly associated to HCQ [36], darunavir/cobicistat [37], and tocilizumab [38].

Cases of episodes of manic behaviour with psychotic features, persecutory delusions, anxiety, and reality detachment triggered by chloroquine were described [39–43]. Considering that HCQ and chloroquine have similar pharmacological properties, their toxicity profiles could be considered comparable. A meta-analysis [44] and a pharmacovigilance study on registry [45] confirmed the association between HCQ and psychiatric events. The mechanisms responsible for the psychiatric ADRs following HCQ exposure are not fully clarified. Among proposed hypotheses, there are the cholinergic imbalance related to the inhibition of acetylcholinesterase, prostaglandin E antagonism, the accumulation of HCQ toxic metabolites in lysosomes, and the down-regulation of glycoprotein-P in the blood–brain barrier [46]. Moreover, HCQ seems to inhibit the serotonin transporter, increasing its levels in the synapsis, and to act as *N*-methyl-d-aspartate agonist and γ-aminobutyric acid antagonist [46]. In general, psychiatric ADRs resolution follows HCQ withdrawal.

Among psychiatric ADRs, only “abnormal dreams” are reported in darunavir/cobicistat summary of product characteristics (SPC) [37], and, to date, the literature is lacking evidence on this topic. In general, protease inhibitors have limited central nervous system penetration, and therefore, less-pronounced neurological and psychiatric ADRs [47]. Among this group, ritonavir alone or in combination is more likely to produce psychiatric ADRs, in particular mood changes, agitation, and anxiety. In a clinical trial, HIV patients were randomised to darunavir/ritonavir or darunavir/ritonavir in combination with two nucleoside/nucleotide reverse transcriptase inhibitors [48]. After 48 weeks of therapy, grade 1–4 nervous system and psychiatric ADRs were seen in 16% and 9% of patients in each treatment arm. Researchers reported the following psychiatric manifestations: anxiety, depression, obsessive–compulsive disorder, and psychotic crisis. Considering that cobicistat is a CYP3A4 inhibitor and recommendations reported in its SPC suggest reducing the dosages of concomitant central nervous system medications, actually, there is no possibility of a drug therapeutic failure of antipsychotics driven by the pharmacokinetic properties of suspected protease inhibitors. Therefore, psychiatric ADRs observed in our sample may have been mainly related to high-dose HCQ and to underlying psychiatric comorbidities.

After a literature search, we ascertained the lack of evidence on psychiatric ADRs related to tocilizumab [38]. Nowadays, the association between tocilizumab and psychiatric ADRs cannot be fully explained. As for protease inhibitors, particularly darunavir/cobicistat, psychiatric ADRs may have been mainly related to high-dose HCQ and to pre-existing psychiatric disorders.

When diagnosis of a psychiatric ADRs is made, the best solution is to discontinue any suspected drug. Based on our clinical experience, depending on psychiatric clinical manifestation and on QT interval values, the administration of specific antipsychotic medications (i.e. chlorpromazine) could be considered. Usually, patient’s mental status reverts to normal in a few days. In case of emergencies, emotional distress is ubiquitous, but some groups may be more vulnerable than others. In particular, people at heightened risk for COVID-19, those who contract the disease, and people with pre-existing medical or psychiatric conditions are at increased risk for adverse psychosocial outcomes [49]. Particular attention should also be given to mental health of people in conditions of increased risk, such as women during pregnancy [50] or post-partum [51].

### Gastrointestinal and hepatic disorders

We observed four cases of GI and hepatic disorders, in particular “nausea”, “vomiting”, “diarrhoea” and “hypertransaminasemia”. In these cases, all classes of COVID-19 medications were involved and GI intolerance often led to pharmacological switching between the associations lopinavir/ritonavir and darunavir/cobicistat. This kind of non-specific ADRs is frequently (common or very common) observed for all medication classes, including that of COVID-19 treatments [30, 36–38, 52]. The evaluation of causality assessment for GI and hepatic disorders must take into consideration the presence of concomitant medications and the underlying SARS-CoV-2 infection, which is commonly associated with GI symptoms [53].

## Conclusion

Despite the small number of patients, the evidence reported in the present analysis confirms that the clinical burden of DDIs in SARS-CoV-2 hospitalised patients is relevant. Moreover, the risk of unexpected and uncommon ADRs, such those referred to psychiatric disorders, was highlighted. In this population, COVID-19 treatments should be used with extreme caution, especially in fragile and polymedicated patients. Although living in the context of a global emergency and looking for an effective therapeutic treatment, drug safety should never be overlooked, especially in the presence of DDIs.

## Supplementary Information

Below is the link to the electronic supplementary material.

Supplementary file1 (DOCX 16 KB)
