# Supplementary material for: Adverse drug reactions in SARS-CoV-2 hospitalised patients: a case-series with a focus on drug–drug interactions
Source: Intern Emerg Med. 2020 Dec 23;16(3):697–710. doi: 10.1007/s11739-020-02586-8 (PMC7755981; doi:10.1007/s11739-020-02586-8)
Supplement: Supplementary file 1 — Supplementary file1 (DOCX 16 KB) [file 11739_2020_2586_MOESM1_ESM.docx]

**Supplementary table 1** - Major and moderate DDIs between concomitant medications reported in patients’ anamnesis

| **Interacting drugs** | **Interacting active principles** | **Interaction severity** | **Interaction effects** |
| --- | --- | --- | --- |
| **CNS medications** | Alprazolam-codeine or morphine | Major | Increased risk of CNS- and respiratory system depression. |
|  | Alprazolam-furosemide | Moderate | Increased risk of hypotension. |
|  | Alprazolam-omeprazole | Moderate | Increased blood levels of alprazolam. |
|  | Alprazolam-trazodone | Moderate | Increased risk of CNS- and respiratory system depression. |
|  | Citalopram-promazine | Major | Increased risk of QT ECG prolongation. |
|  | Citalopram-quetiapine | Major | Increased risk of QT ECG prolongation. |
|  | Clonazepam-dexmedetomidine | Moderate | Increased risk of CNS- and respiratory system depression. |
|  | Clonazepam-spironolactone | Moderate | Increased risk of hypotension. |
|  | Clonazepam-propofol | Moderate | Increased risk of CNS- and respiratory system depression. |
|  | Clonazepam-risperidone | Moderate | Increased risk of CNS- and respiratory system depression. |
|  | Codeine-morphine | Major | Increased risk of CNS- and respiratory system depression. |
|  | Codeine-trazodone | Moderate | Increased risk of serotonin syndrome. |
|  | Codeine-valproic acid | Moderate | Increased risk of CNS- and respiratory system depression. |
|  | Dexmedetomidine-amlodipine | Moderate | Increased risk of hypotension. |
|  | Dexmedetomidine-midazolam | Moderate | Increased risk of CNS- and respiratory system depression. |
|  | Dexmedetomidine-olanzapine | Moderate | Increased risk of CNS- and respiratory system depression. |
|  | Dexmedetomidine-propofol | Moderate | Increased risk of CNS- and respiratory system depression. |
|  | Dexmedetomidine-ramipril | Moderate | Increased risk of hypotension. |
|  | Dexmedetomidine-risperidone | Moderate | Increased risk of CNS- and respiratory system depression. |
|  | Dexmedetomidine-spironolactone | Moderate | Increased risk of hypotension. |
|  | Haloperidol-verapamil | Moderate | Increased risk of hypotension. |
|  | Lorazepam-bisoprolol | Moderate | Increased risk of hypotension. |
|  | Lorazepam-furosemide | Moderate | Increased risk of hypotension. |
|  | Lorazepam-haloperidol | Moderate | Increased risk of CNS- and/or respiratory-depressant effects. |
|  | Lorazepam-olanzapine | Major | Increased CNS- and CV-depressant effects (i.e., low blood pressure, weak pulse, muscle weakness, drowsiness, and dizziness). |
|  | Lorazepam-risperidone | Moderate | Increased CNS- and/or respiratory-depressant effects may be additively or synergistically. |
|  | Lorazepam-tamsulosin | Moderate | Increased risk of hypotension. |
|  | Midazolam-propofol | Moderate | Increased risk of CNS- and respiratory system depression. |
|  | Midazolam-spironolactone | Moderate | Increased risk of hypotension. |
|  | Morphine-trazodone | Moderate | Increased risk of CNS- and respiratory system depression. |
|  | Olanzapine-amlodipine | Moderate | Increased risk of hypotension. |
|  | Olanzapine-bisoprolol | Moderate | Increased risk of hypotension. |
|  | Olanzapine-citalopram | Moderate | Increased risk of QT ECG prolongation. |
|  | Olanzapine-clonazepam | Major | Increased risk of CNS depression. |
|  | Olanzapine-midazolam | Major | Increased risk of CNS depression. |
|  | Olanzapine-promazine | Moderate | Increased risk of paralytic ileus, hyperthermia, heat stroke, and anticholinergic intoxication syndrome. |
|  | Olanzapine-propofol | Moderate | Increased risk of QT ECG prolongation. |
|  | Olanzapine-quetiapine | Moderate | Increased risk of paralytic ileus, hyperthermia, heat stroke, and anticholinergic intoxication syndrome. |
|  | Olanzapine-spironolactone | Moderate | Increased risk of hypotension. |
|  | Promazine-bisoprolol | Moderate | Increased risk of hypotension. |
|  | Promazine-quetiapine | Moderate | Increased risk of QT ECG prolongation. |
|  | Quetiapine-bisoprolol | Moderate | Increased risk of hypotension. |
|  | Risperidone-amlodipine | Moderate | Increased risk of hypotension. |
|  | Risperidone-olanzapine | Moderate | Increased risk of paralytic ileus, hyperthermia, heat stroke, and anticholinergic intoxication syndrome. |
|  | Risperidone-midazolam | Moderate | Increased risk of CNS- and respiratory system depression |
|  | Risperidone-propofol | Moderate | Increased risk of QT ECG prolongation. |
|  | Risperidone-spironolactone | Moderate | Increased risk of hypotension. |
|  | Sertraline-furosemide | Moderate | Increased risk of hyponatraemia. |
|  | Sertraline-loperamide | Moderate | Increased risk of QT ECG prolongation. |
|  | Sertraline-lorazepam | Moderate | Increased CNS- and/or respiratory-depressant effects may be additively or synergistically. |
|  | Sertraline-olanzapine | Moderate | Increased risk of QT ECG prolongation. |
|  | Sertraline-risperidone | Moderate | Increased risk of QT ECG prolongation. |
|  | Sertraline-tamsulosin | Moderate | Increased blood levels of tamsulosin due to sertraline inhibition of CYP450 3A4 and/or 2D6 and increased risk of hypotension. |
|  | Trazodone-citalopram | Major | Increased risk of serotonin syndrome and of QT ECG prolongation. |
|  | Valproic acid-clonazepam | Moderate | Increased risk of drowsiness and seizures. |
|  | Valproic acid-haloperidol | Moderate | Increased risk of CNS- and/or respiratory-depressant effects. |
|  | Valproic acid-dexmedetomidine | Moderate | Increased risk of CNS- and/or respiratory-depressant effects. |
|  | Valproic acid-morphine | Moderate | Increased risk of CNS-depressant effects. |
|  | Valproic acid-olanzapine | Moderate | Increased risk of hepatic toxicity. |
|  | Valproic acid-propofol | Moderate | Increased blood levels of propofol. |
|  | Valproic acid-risperidone | Moderate | Increased blood levels of valproic acid. |
|  | Valproic acid-trazodone | Moderate | Increased risk of CNS- and/or respiratory-depressant effects. |
| **CV medications** | Acetylsalicylic acid-amlodipine | Moderate | Increased risk of hypertension. |
|  | Acetylsalicylic acid-enoxaparin | Major | Increased risk of bleeding. |
|  | Acetylsalicylic acid-betamethasone or methylprednisolone | Moderate | Reduced serum concentrations and therapeutic effects of salicylates. |
|  | Acetylsalicylic acid-candesartan | Moderate | Increased risk of candesartan inefficacy due to acetylsalicylic acid-related inhibition of renal prostaglandin synthesis. |
|  | Acetylsalicylic acid-citalopram | Moderate | Increased risk of bleeding. |
|  | Acetylsalicylic acid-losartan or valsartan | Moderate | Increased risk of losartan inefficacy due to acetylsalicylic acid-related inhibition of renal prostaglandin synthesis. |
|  | Acetylsalicylic acid-sertraline | Moderate | Increased risk of bleeding. |
|  | Acetylsalicylic acid-verapamil | Moderate | Increased blood levels of valproic acid. |
|  | Acetylsalicylic acid-verapamil | Moderate | Increased risk of bleeding, bruising, headache, dizziness, weakness. |
|  | Amiodarone-furosemide | Major | Increased risk of QT ECG prolongation. |
|  | Amiodarone-warfarin | Major | Increased risk of bleeding. |
|  | Atorvastatin-amiodarone | Moderate | Increased blood levels of atorvastatin and increased risk of myopathy. |
|  | Atorvastatin-bicalutamide | Moderate | Increased blood levels of atorvastatin and increased risk of myopathy. |
|  | Atorvastatin-pantoprazole | Moderate | Increased blood levels of atorvastatin and increased risk of liver damage and myopathy due to competitive inhibition of intestinal P-glycoprotein and CYP450 3A4. |
|  | Bisoprolol-alprazolam | Moderate | Increased risk of hypotension. |
|  | Bisoprolol-gliclazide | Moderate | Increased risk of hypoglycaemia. |
|  | Bisoprolol-furosemide | Moderate | Increased risk of hyperglycaemia and hypertriglyceridemia. |
|  | Bisoprolol-linezolid | Moderate | Increased risk of hypotension, orthostasis, bradycardia, and heart failure. |
|  | Bisoprolol-methylprednisolone | Moderate | Increased risk of bisoprolol inefficacy due to corticosteroids-related sodium and fluid retention. |
|  | Candesartan-enoxaparin | Moderate | Increased risk of hyperkalaemia. |
|  | Candesartan-betamethasone | Moderate | Increased risk of candesartan inefficacy due to corticosteroids-related sodium and fluid retention. |
|  | Clopidogrel-atorvastatin | Moderate | Decreased efficacy of clopidogrel due to a mechanism of competitive inhibition of CYP450 3A4. |
|  | Clopidogrel-citalopram | Moderate | Increased risk of bleeding. |
|  | Losartan-betamethasone | Moderate | Increased risk of losartan inefficacy due to corticosteroids-related sodium and fluid retention. |
|  | Olmesartan-potassium chloride | Major | Increased risk of hyperkalaemia. |
|  | Perindopril-furosemide | Moderate | Increased risk of hypotension and hypovolemia. |
|  | Perindopril-glicazide | Moderate | Increased risk of hypoglycaemia. |
|  | Perindopril-insulin | Moderate | Increased risk of hypoglycaemia. |
|  | Perindopril-metformin | Moderate | Increased risk of hypoglycaemia. |
|  | Perindopril-vildagliptin | Moderate | Increased risk of angioedema. |
|  | Ramipril-allopurinol | Major | Increased risk of severe hypersensitivity reactions, neutropenia, agranulocytosis, and serious infections. |
|  | Ramipril-clonazepam | Moderate | Increased risk of hypotension. |
|  | Ramipril-olanzapine | Moderate | Increased risk of hypotension. |
|  | Ramipril-risperidone | Moderate | Increased risk of hypotension. |
|  | Ramipril-spironolactone | Major | Increased risk of hyperkalaemia. |
|  | Ramipril-vildagliptin | Moderate | Increased risk of angioedema. |
|  | Warfarin-amoxicillin/clavulanate | Moderate | Increased risk of bleeding. |
|  | Warfarin-rabeprazole or pantoprazole | Moderate | Increased risk of bleeding. |
| **Other interactions** | Fluticasone propionate/vilanterol-linezolid | Moderate | Increased risk of hypertension, palpitation, tachycardia, and chest pain. |
|  | Furosemide-ceftriaxone | Moderate | Increased risk of nephrotoxicity. |
|  | Furosemide-gliclazide | Moderate | Increased risk of hyperglycaemia. |
|  | Furosemide-insulin | Moderate | Increased risk of hyperglycaemia. |
|  | Furosemide-linezolid | Moderate | Increased risk of hypotension and orthostasis. |
|  | Furosemide-methylprednisolone | Moderate | Increased risk of hypokalaemia. |
|  | Furosemide-pantoprazole or esomeprazole | Moderate | Increased risk of hypomagnesemia. |
|  | Furosemide-vildagliptin | Moderate | Increased risk of hyperglycaemia. |
|  | Gliclazide-furosemide | Moderate | Increased risk of hyperglycaemia. |
|  | Insulin-gliclazide | Moderate | Increased risk of hypoglycaemia. |
|  | Insulin-vildagliptin | Moderate | Increased risk of hypoglycaemia. |
|  | Vildagliptin-gliclazide | Moderate | Increased risk of hypoglycaemia. |

CNS: central nervous system; CV: cardiovascular; ECG: electrocardiogram.
